# Physiological responses of pigs to preslaughter handling: infrared and thermal imaging applications

**DOI:** 10.1080/23144599.2020.1821574

**Published:** 2020-09-27

**Authors:** Salvador Flores-Peinado, Daniel Mota-Rojas, Isabel Guerrero-Legarreta, Patricia Mora-Medina, Rosy Cruz-Monterrosa, Jocelyn Gómez-Prado, María Guadalupe Hernández, Jesús Cruz-Playas, Julio Martínez-Burnes

**Affiliations:** a Neurophysiology, Behavior and Assessment of Welfare in Domestic Animals, DPAA. Universidad Autónoma Metropolitana (UAM), Mexico City, Mexico; b Department of Biotechnology, Emeritus Professor Universidad Autónoma Metropolitana-Iztapalapa, (UAM-I), Mexico City, Mexico; c Livestock Science Department, Universidad Nacional Autónoma de México (UNAM), Facultad de Estudios Superiores Cuautitlán, State of Mexico, Mexico; d Department of Food Science. Universidad Autónoma Metropolitana, Lerma, Mexico; e Graduate and Research Department, Faculty of Veterinary Medicine, Universidad Autónoma de Tamaulipas, Victoria City, Mexico

**Keywords:** Pig welfare, transport, abattoir, skin temperature, infrared thermography

## Abstract

Loading, transport, unloading, lairage and stunning are the principle *ante-mortem* events that generate negative responses associated with stress in pigs. For this reason, it is important to verify the condition of animals throughout the supply chain in order to ensure their welfare and obtain, at the end of the slaughtering process, high-quality carcases and meat. Several studies have identified the indicators and samples that need to be taken into account to properly measure and evaluate the responses that these animals emit to the stressors involved. However, these procedures must be carried out quickly and by non-invasive means so as not to impede the flow of animals through the operations of loading, transport, unloading and lairage. Therefore, the objective of this review article is to analyse the stressful events that pigs experience during these events and discuss the use of the infrared thermography (IRT) as an alternative tool for measuring stress based on temperature changes on the surface of pigs’ skin. We argue that infrared thermography can be used as a strategy to improve animal welfare during loading, transport, unloading and lairage by preventing fatigue and deaths, and decreasing negative impacts on meat quality, such as pale, soft and exudative (PSE), or dark, firm and dry (DFD) conditions.

## 1. Introduction

**Figure 1.**
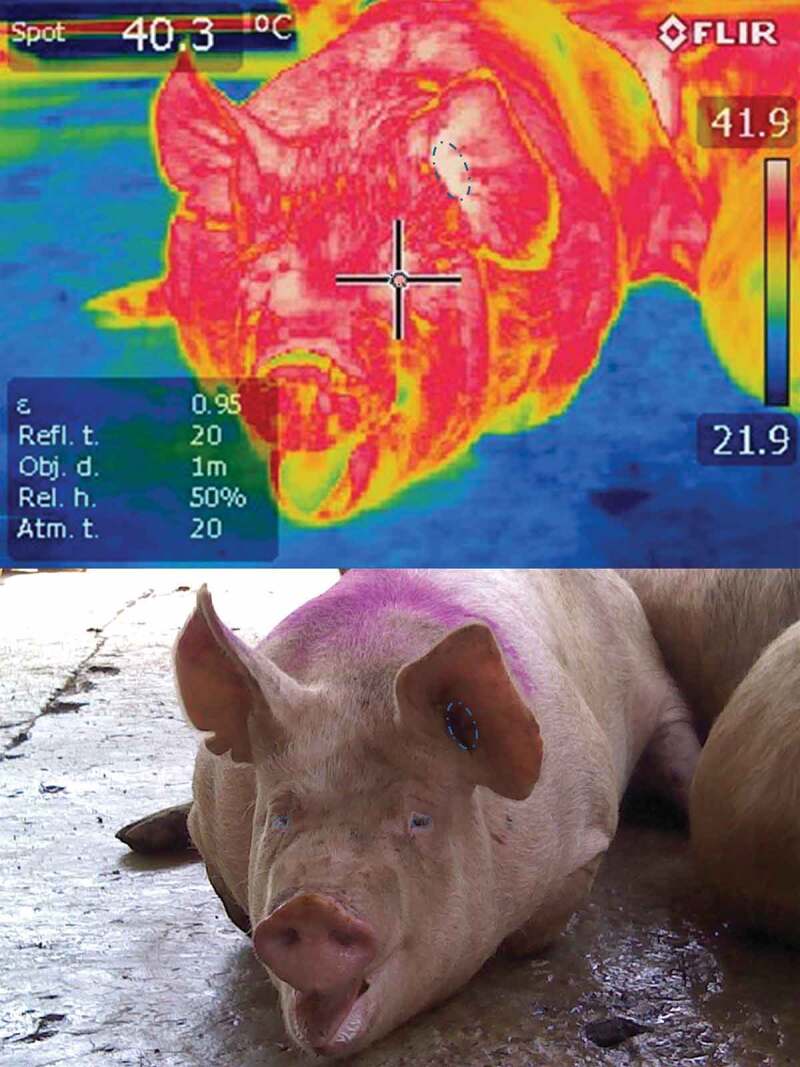
A pig with hyperthermia upon arrival at an abattoir

^24^,^67^,^78^,^84^,^90^

The handling of pigs during the processes of loading, transport, unloading, lairage and stunning are identified as the principle *ante-mortem* events that generate negative responses associated with stress [1–3]. These operations expose pigs to diverse stress factors that may be psychological (social mixing, overcrowding, fights, human-animal contact, exposure to novel surroundings) or physical (hunger, thirst, fatigue, lesions, extreme temperature changes) [4–6]. In proportion to their duration and intensity [7–10] these stressors can severely impact animal welfare by increasing the incidence of cutaneous lesions [7] and deteriorating meat quality. Stress in animals is a nervous system response to diverse environmental stimuli. The nervous system’s release of the adrenocorticotropic hormone (ACTH) generates the release of catecholamines that prepare the organism for a “fight-or-flight” response, followed by the release of adrenal glucocorticoids, which act on energy metabolism [11]. Any factor that fosters an increase in the metabolic rate exerts an adverse effect on commercial pigs, a species of animal that is highly-susceptible to hyperthermia [12] (Figure 1) because it cannot efficiently dissipate heat, so this accumulates in internal temperature, that is, in abdominal and thoracic cavities (body temperature increases 1 or ≥2.5°C) [13,14]. Depending on the severity of hyperthermia, hypoxia may occur in peripheral tissues (The partial pressure of carbon dioxide increases., ↓pO_2_ levels, dyspnoea, panting) [15]. An increase in pCO_2_ in the blood raises the level of glycolysis (blood glucose increases) resulting in lactate accumulation [9,14,15]. In pigs that are especially susceptible to this condition these events can produce hypoxia in peripheral tissues [15] and, in extreme cases, pork stress syndrome and death due to acute metabolic acidosis [14,15].

All these metabolic and physiological changes can be detected through a very useful tool which is the infrared thermography (IRT) [16,17]. The infrared thermal camera is a device that detects microcirculatory variations in the skin [17] that result from metabolic changes [16] in pigs transported to the abattoir. This could be a predictor of imbalances in post-mortem biochemical changes that could affect meat quality traits. For these reasons, it is important to identify the degree of stress that pigs suffer. Various tools have been proposed for this objective: evaluating behavioural modifications such as aggression [18] or abnormal movements (fatigue, muscular tremor) [14,18], and measuring physiological alterations in fattening pigs by determining such blood metabolites as serum concentrations of haptoglobin (Hp), C-reactive protein (CRP), and cortisol, or pH, lactate and glucose levels [9]. A more recent technique employs IRT to assess animal welfare by evaluating organisms’ responses to the stressors that can affect the level of productivity (eggs, milk or meat). Thermography functions by detecting and quantifying surface temperatures in the most external millimetres of the skin, which are dependent on blood circulation [19]. Body temperature changes constantly throughout the day due to several factors, including the physical activity of the organism, its surroundings, climatological conditions, and the possible presence of an infectious process [19,20].

Therefore, it is of fundamental importance to verify the surface temperature of pigs in a fast, non-invasive manner immediately upon their arrival at the abattoir, for hyperthermia in pigs affects not only their level of welfare, but also has direct adverse consequences that can deteriorate meat quality. In light of this brief discussion, the aim of this review article is to analyse the stressful events that pigs experience during loading, transport, unloading and lairage at the abattoir, and discuss the use of the IRT as a tool for measuring stress throughout the pork supply chain by detecting changes in pigs’ surface temperature.

## 2. Stressors and facilities in fattening pigs from loading to the abattoir and their consequences

Among the events that compromise the welfare of animals destined for human consumption, transport and lairage prior to slaughter, together with stunning, are considered three stress factors that can severely deteriorate the health and overall welfare of animals and, therefore, have negative effects on meat quality [8,9,21]. Other negative events observed during these operations are injuries, bruising, fractures, weight loss and death as a consequence of problems associated with animal welfare [21,22]. Due to these circumstances, ensuring the welfare of commercialized animals now constitutes a global problem that affects every sector of the food industry, obliging producers and researchers to search for ways to improve production practices and the norms of international commerce to satisfy consumer demand for foods of optimum quality [23–25].

### 2.1. Loading ramps

Each phase of transport – loading, the journey itself, unloading – may involve activities that provoke stress in pigs that increases their heart rate, especially if handling procedures are inadequate. Examples include loading ramps with slopes >20° over the horizontal [26] because they make it difficult for pigs to move, and cause falls or slipping. Above all, they can instil fear in the group that impedes it from continuing its forward movement [22,25,27].

### 2.2. Vehicle characteristics

The type of floor in the transport vehicle is another factor that can increase stress in pigs and affect meat quality due to the strenuous effort required to avoid slipping, which triggers muscular thermogenesis that can exhaust glycogen reserves [28,29]. Guàrdia et al. [30] sustain the importance of assessing the type of flooring in light of observations which show that a polyester base reduces the risk of pale, soft and exudative muscle (PSE) by around 1.5% by offering a more comfortable environment for transport that reduces noise and slippage due to its soft, wrinkled texture. A polyester base also has much better thermal insulating properties than aluminium or iron, which tend to generate high incidences of PSE meat. In a later study, Guàrdia et al. [28] determined that the incidence of the dark, firm and dry (DFD) defect in pork was higher in pigs transported in trucks with metal flooring than in vehicles with aluminium floors (8.38 vs. 3.31%). They further mentioned that metal-iron is not the most suitable material for flooring because pigs slip and have difficulty standing firmly on that material, generating harmful stress. Finally, the use of hydraulic systems with sloped loading ramps has been found to reduce the incidence of PSE meat [28,30].

### 2.3. Time and distance travelled

The length of the journey in pigs is another stressor associated with transport that can impact animal welfare, which can vary from short jaunts to hundreds of kilometres [22,23]. Any period of transport exposes animals to factors that can cause physical and mental stress. Regarding journey duration, Mota-Rojas et al. [22] concluded that longer transport times increase the incidence of lesions to the skin and subcutaneous and muscular tissues, and produce signs of fatigue and hyperventilation in animals. Furthermore, even pigs transported over short distances tend to have higher incidences of PSE due to increased body temperatures (>40°C) [14], while the meat of those carried over long distances tends to have more DFD defects [5,31–33]. In this vein, Gosálvez et al. [34] reported that pig mortality increases with greater distance (0.21% on journeys <50 km; 0.46% on journeys >100 km). Warriss et al. [35] and Warriss et al. [36] obtained similar results, but Haley et al.’s report [37] disagreed, as they found that for each 50-km increase in distance, mortality decreased by only 0.03%. Other observations have shown that pigs transported for less than 30 minutes in summertime are more aggressive and difficult to handle, and that under those conditions the incidence of lesions and PSE meat increases [6,7,38]. The work by Sutherland et al. [39] found that pre-slaughter transport and lairage times are factors that markedly affect pig mortality due to the excessive metabolic response. In a IRT study in slaughter pigs at the abattoir conducted by Warris et al. [40], a significant correlation (r = 0.71, P < 0.001) was found between the ear temperature of pigs measured in the pen at the blood temperature that the animals lost during exsanguination.

### 2.4. Loading density

Directive 95/29/CE [41] for the protection of animals during transport stipulates that pigs must have sufficient room to lie down and stand up in natural positions. To satisfy these minimal requirements, the loading density of pigs weighing 100 kg cannot exceed 235 kg/m^2^; that is, an available space of 0.425 m^2^/100 kg of animal weight. In this regard, Guàrdia et al. [28] mentioned that a space of 0.42 m^2^ for a 100-kg pig is only appropriate on journeys longer than 3 h, because overloading and the consequent, drastic reduction of space can generate problems like crushing, overcrowding and fighting during transport [42]. One recommendation is to increase the space available in the vehicle by 10% during hot periods, especially if heavier pigs are being transported, since they are more susceptible to heat stress because they produce more body heat (+2% for every additional 5 kg of live weight) [43,44] but have a limited capacity to dissipate that excess heat. This also applied if the transport route runs through heavy traffic areas or urban zones where ventilation may decrease due to lower speeds [45]. Studies by Cobanovic et al. [46] found that reduced space (<0.3 m^2^/100 kg pig) during transport increased indices of lesions, final pH values, and the incidence of DFD meat. However, the same study [46] demonstrated that when more space is available (above 0.5 m^2^/100 kg), pigs can move around more freely inside the vehicle, resulting in an increased frequency of cutaneous lesions, which can serve as an important tool for predicting defects in meat quality since higher incidences of such injuries have been associated with higher percentages of PSE meat [46].

### 2.5. Vibrations

Vibrations along transport routes also alter pigs’ physiological responses. The study by Perremans et al. [47] utilized the heart rate of in-transit pigs as an indicator of animal welfare. They observed that the effect of vibrations modified the animals’ heart rate values because of the enormous energy expenditures required to maintain their balance. This study also suggested that the harmful stress attributed to vibrations may be greater than that which occurs during loading and unloading to and from the vehicle [5]. In addition, vibration during transport can induce stress and travel sickness in pigs [6]. Bradshaw et al. [48] demonstrated an increase in plasma lysine vasopressin concentrations in pigs, the hormone that regulates the release of adrenocorticotropic hormone (ACTH), which generates secretion of corticosteroids and, in turn, fosters vasoconstriction [33]. Lysine vasopressin is also associated with movement-induced dizziness, manifested by the symptoms of nausea and vomiting and by such behaviours as retching, chewing, foaming at the mouth and sniffing the air while standing [48]. Hence, one recommendation is to eliminate low-frequency vibrations and abrupt accelerations because those actions increase the heart rate and the consequent blood flow in pigs during transport. Vibration-induced stress will be reduced as more vehicles with pneumatic suspension come to be utilized [49].

### 2.6. Climatic conditions of the journey

It is important to keep in mind that pigs are particularly sensitive to high ambient temperatures, especially if other mechanisms for dissipating heat, such as peripheral vasodilatation [15,50], are compromised as a result of stress responses. This is because pigs have very few functional sweat glands, so their capacity to dissipate heat via sweating is virtually nil [50,51]. As a result, transporting pigs under conditions of high ambient temperature (>35°C), as in the summer season in some South American countries, tends to increase the incidence of PSE meat, compared to wintertime transport. Guàrdia et al. [28] mentioned that the period of the year strongly influences the incidence of DFD meat, which is higher in winter than summer (7.6 vs. 6.07%), likely because the cold weather causes the pigs to (i) shiver, which accelerates the exhaustion of glycogen reserves in their muscles; and (ii) huddle together, which can cause fighting [34], more often among females than males (8.06 vs. 5.01%) [28]. Observations also show that the risk of the incidence of PSE meat in summer is almost double that of winter (6.50 vs. 3.40%) because of pigs’ sensitivity to high temperatures and difficulty in dissipating body heat (Figure 1). The digital image in Figure 1 shows no signs of erythematosus, but the thermographic image reveals white areas that represent temperatures above 40°C, especially around the left ear and the periocular area. As a consequence of this hyperthermic condition, the pig suffered dyspnoea caused by the biochemical processes of hypercapnia and respiratory and metabolic acidosis. In addition, the risk of presenting the PSE condition is 0.50% higher in males than females [52], an effect due to differences between the sexes in the use of glycogen during transport, and the composition of muscle fibres [53]. Regarding the effect of seasonality on pig mortality, available information is inconclusive. A study by Gosálvez et al. [34] analysed 496 journeys to the same abattoir to determine the influence of season (spring, summer, autumn, winter), distance travelled (<50 km, 50–100 km, >100 km), and number of farms (one or more), on the transport of pigs in commercial conditions in Spain. For some of the physical and in-canal parameters, they reported that mortality was not affected by season, though several other authors affirmed that the number of deaths during transport increases under hot climatic conditions [54,55]. These results could reflect that – at least in Spain – handlers take extra precautions in hotter seasons; for example, night-time travel, lower load density, and providing sprinklers [56]. This could explain the low percentages of condemned (0.08%) and culled (0.29%) cadavers in summertime. Their findings led them to conclude that adequate development of the transport system and suitable handling – at least under commercial conditions in Spain – can reduce the effects of seasonality on pig welfare, though the mixing of unfamiliar animals may still be harmful.

### 2.7. Rest during the journey

It is important to note that rest intervals during long journeys may impact pig welfare negatively. As Rioja-Lang et al. [57] reported, when the maximum allowable travel time is exceeded, pigs must be unloaded and led into pens where they can rest while receiving food and water. Rest periods are scheduled to allow the pigs to recover from the effects of dehydration, hunger and fatigue before continuing the journey. However, scientific studies have shown that the stress caused by unloading and loading in the rest area – reflected in increased heart rates [58] – together with that generated by mixing pigs in a new environment may actually be harmful for their welfare [59]. Hence, it has been suggested that it is better to feed and hydrate the pigs while they are inside the vehicle during long journeys, to avoid this stressful handling and its risks for biosecurity.

### 2.8. Unloading

The combination of various stressors is likely to have a cumulative effect [60], so unloading is another stress factor that must be considered. The meat quality of fattening pigs is affected by increases in muscular temperature [15] due to stress begins with fasting on the farm and continues through loading, transport and, finally, the forced descent from the vehicle down an inclined ramp which demands substantial muscular exercise that can increase the pigs’ heart rate. Unloading is another stress factor that must be considered, in addition to loading and transport. In fact, meat quality is affected by stress factors that begin with fasting in corrals on the farm and continue through loading, transport and, finally, the forced descent from the truck down an inclined ramp [22,25,31]. In addition, observations of this stage of the process showed that some stressed, fatigued fattening pigs are reluctant – or refuse – to move during or just after unloading at the abattoir, even though they can stand up. These pigs present one or more of the following physiological states: increased body temperature, difficulty breathing, trembling, patchy skin discoloration, recumbency, and in some cases death [60]. Thus, quantifying the behaviour of animals during unloading is another good indicator of welfare. Protocols developed in the framework of the Welfare Quality for Pigs Project [61] assess two behaviours related to this phase of handling. First, they count the animals that back up during unloading; that is, the pigs that upon reaching the chute leading to the lairage pens seek to turn back and return to the vehicle because of the novel environment or poor handling, or simply because this is a normal behaviour for some. This behaviour may occur in anywhere from 0.47 to 18.69% of the pigs that reach the chute [62]. The second aspect involves unloading processes that are too hurried, where the personnel in charge have insufficient time to carefully guide the animals into the holding pens [62,63]. Under those conditions, pigs may also baulk and quickly fill the chute. If handlers move to the back of the pack to try to prod the pigs forward, the animals tend to turn around, collide with one another, and/or flee wildly, thus increasing stress levels and causing the mixing of unfamiliar animals [62,63].

Another reliable indicator of the degree of stress in pigs consists in quantifying their vocalizations during *ante-mortem* processes. Vermeulen et al. [64], for example, observed 8,508 pigs at 18 different abattoirs during 4 phases: just before unloading, during unloading, in the lairage pens, and through the chute leading to the stunning chamber. Findings showed that the percentage of pigs that vocalized and the mean sound level produced just before, and then during unloading, significantly affected the percentage of PSE meat, because the acute sounds that pigs produce increase stress and activate defence mechanisms [65] thus accelerating the rate of glycolysis after death. Later, the absence of oxygen generates lactic acid and decreases pH. In addition, high *post-mortem* body temperatures generate high rates of muscle protein denaturalization [66–68]. Vermeulen et al. [64] found that pH was adequate in the *Longissimus thoracis* muscle (pH 6.21) with a reduction in the percentage of PSE meat only when good handling practices were applied during unloading. However, they concluded that while each *ante-mortem* stage impact the quality of the meat produced, the phases closest to stunning are most critical in terms of developing PSE meat. Those researchers suggested that improving pre-slaughter conditions will ensure the welfare of the pigs and good meat quality [66–68].

### 2.9. Ante-mortem lairage in corrals

The lairage period at the abattoir is extremely important because it can have a negative impact on pig welfare, but can also cause economic losses along the supply chain. Errors committed in pre-slaughter stages can cause irreversible defects in carcase or meat quality that nullify efforts by productive sectors to increase yields and ensure animal welfare [31]. Exposing pigs to various adverse conditions (stressors), such as lack of food and water, real or potential threats and danger, hunger, mixing of animals from different origins, lack of thermal comfort (extreme heat or cold), light, and space restrictions during lairage can exacerbate the effects of previous stages. Lairage period of 2–4 h is recommended, though this may vary with the conditions in different abattoirs [31]. Establishing an ideal antemortem lairage period is necessary because pigs need to recover their physiological balance and ensure the adequate transformation of muscle-to-meat. Lairage should foster both animal welfare and the procurement of better-quality meat. While it is true that diverse factors can compromise the welfare of pigs during repose, handlers should avoid mixing animals from different groups, as this measure can reduce fighting during lairage by up to 50%. Fasting times for pigs should never exceed 12 hours, so when this occurs they must be given food to prevent depletion of their energy reserves, as this will alter their energy metabolism, acid–base balance, hydric balance, and gas exchange and, hence, reduce the quality of the meat obtained [1,25].

Lairage in corrals at the abattoir prior to slaughter is believed to give animals the time and conditions they require to recover from earlier handling and, consequently, ensure good quality meat [69]. By minimizing the stress factors associated with transport and restoring glycogen reserves, lairage reduces the incidence of undesirable characteristics in meat [36,70,71], but it presents a significant problem: the time involved.

In some Latin American countries, animals stay in pens for 24 h or more (minimum 12) without food or water before stunning [31]. Such prolonged fasting is not recommended because energy reserves in the animals’ muscles can only be re-established through gluconeogenesis. Under these fasting conditions, the absence of food and water catabolizes body fat and protein, producing such negative effects as weight loss, low yields in carcase, and poor meat quality [72]. Costa, et al. [73] found that animals that do not fast tend to fight more frequently and for longer times during lairage. In another study, Costa et al. [74] showed that a long lairage period (>14 hours) – or even a 3-h period of repose [74] – compromises animal welfare, as evidenced by high blood lactate concentrations and a higher frequency of skin lesions. Short periods (<3 h) are often characterized by increased fighting and aggressiveness due to the catecholamines or cortisol released as a result of the dissipation of muscular heat that increases muscular temperature. These conditions cause an increase in the incidence of PSE meat [69]. Other studies affirmed that the risk of producing DFD meat increases by 11.6% with lairage times above 3 h, while stabling for 9 h increases this defect by 18.6%. Guàrdia, et al [28] suggested that night-time lairage periods can increase the incidence of DFD meat by as much as 24.9%, likely because the animals use their glycogen-based energy reserves for thermoregulation as they seek to raise their body temperature under cold conditions. Those circumstances reduce production of the lactic acid required to maintain the level of acidity for the normal transformation of muscle-to-meat while also increasing pH, which fosters the presentation of DFD meat [28].

Some scientific evidence recommended a lairage period of 2–4 h, though this may well vary depending on the physical conditions and practices at each abattoir [31,75,76]. Total fasting time contributes to higher occurrences of DFD meat, the proportion of DFD meat decreased in animals that experienced total fasting conditions for 14–22 hours, so this period could be recommendable [28]. With respect to blood cortisol levels, results to date are inconclusive because, unlike the findings reported by some authors [77–79]. Costa et al. [74] evaluated 960 pigs from 8 farms in Brazil, finding that blood cortisol levels were not significantly affected by season, fasting time or lairage. These findings, however, may reflect good environmental conditions and correct handling. In any case, the conditions in which lairage is performed modify thermoregulation in pigs. Costa et al. [74] detected higher lactate levels in summertime due to the susceptibility to heat stress of fattening pigs during lairage [80,81]. Efforts to reduce caloric stress in pigs during lairage include the recommendation to provide showers in the summer months that cast large drops followed by a fine mist. The drops fall quickly to humidify the pigs, while the fine mist hangs in the air to increase ambient humidity and foster thermal equilibrium [2]. Wetting the pigs with water post-transport offers three advantages: it refreshes them, reduces cardiovascular expenditure, and helps reduce aggressiveness. In addition, it cleans their skin, thus decreasing the risk of contamination during slaughter [2,77,82].

During *ante-mortem* lairage, pigs must also have potable water available *ad libitum* to achieve correct exsanguination and so obtain meat with desirable characteristics [22]. During this rest time, pigs may recover 1% or more of the weight lost during transport, but fasting facilitates evisceration and reduces the risk of contamination. Prolonged fasting without water, however, could result in weight loss – live and in-canal – due to dehydration [31]. Another important consideration is that a stomach full of water after prolonged fasting [83–85] could endanger meat safety if a laceration occurs.

Some authors argue that it is preferable to slaughter animals (especially bovines) immediately upon arrival at the abattoir because many installations lack the minimum installations required for animals to recover from transport and prior handling [31]. However, slaughtering pigs immediately after unloading increases the likelihood of undesirable or poor quality meat due to stress or fatigue. Under these conditions, high muscular temperatures permit the short-term accumulation of lactic acid that alters the functionality of muscle proteins (actin and myosin) and increases the proportion of PSE meat [1,3,10]. Alarcón-Rojo and Duarte-Atondo [2] recommended that handling in the stages prior to slaughter should reduce harmful stress as much as possible, especially in lairage pens. This can be achieved by improving communications between producers and abattoir operators in order to better plan the logistics of these phases [74].

In this regard, Silva et al. [86] evaluated that the degree of harmful stress before slaughter in pigs under adverse conditions is one factor responsible for the PSE defect in meat. At 24 h after death, PSE and RFN meat (red, firm, not exudative) the latter is the type with optimal quality parameters maintained pH values between 5.8 and 6.3. It is important to note that pH at 24 h after death is determinant in the final characteristics of pork because by that time most of the organism’s biochemical processes have finalized. These authors observed that pigs with 2 h of lairage before sacrifice had pH values closer to the normal range with higher values for their water-retaining capacity (WRC). They further determined that pigs with 2–4 h of *ante-mortem* lairage had colour parameters close to reference values. This body of evidence led them to suggest that reducing the incidence of PSE meat under their study conditions required an optimal *ante-mortem* lairage period of 2 h.

It is, therefore, necessary to establish an ideal *ante-mortem* lairage period because pigs need to recover their physiological equilibrium to ensure the adequate transformation of muscle-to-meat. Lairage should foster both animal welfare and the procurement of better-quality meat. While it is true that diverse factors can compromise the welfare of pigs during repose, handlers should avoid mixing animals from different groups, as this measure can reduce fighting by up to 50%. Fasting times for pigs should never exceed 12 hours, so when this occurs they must be given food to prevent depletion of their energy reserves, as this will alter their energy metabolism, acid–base balance, hydric balance and gas exchange and, hence, reduce the quality of the meat obtained [22,25,31].

### 2.10. Other stressors

Any situation during the pork production chain that generates stress [87,88] must be avoided or, at least, controlled due to the repercussions on animal welfare and meat quality. This includes minimizing speed variations due to abrupt accelerations and decelerations [89] which increase tremors and muscular temperature. Poor road conditions, mixing animals from different farms or pens in the abattoir, establishing new hierarchies, resistance to environmental conditions such as relative humidity and high temperatures [7,22,32,34,89–91], fasting, exercise due to physical effort (remaining standing inside a vehicle in movement), deterioration of social groups, human handling (e.g. loading and unloading), and unfamiliar environmental factors (odours, installations) or novel events can all produce stress with negative consequences for welfare and meat quality.

Stress caused by the social mixing of unfamiliar individuals, for example, can induce aggressive behaviour, though even familiar pigs may fight when exposed to novel events. Aggressiveness is often caused by long fasting periods, though this is exacerbated in intact males compared to castrated males and females. Aggressive behaviour manifested in fighting increases the incidence of skin lesions and, when severe, reduces the value of the meat obtained. Moreover, agonistic behaviours that lead to fighting increase blood lactate and cortisol levels and reduce glycogen reserves in the muscles, raising the meat’s pH value after 24 h [69].

## 3. Vascular changes due to thermoregulation and other physiological responses

In the case of pigs, the physical activity produced in the stages prior to arrival at the abattoir contribute to generating heat due to energy expenditures in contracting muscle fibres (approximately 75% of this energy is converted into heat), and the processes of re-synthesizing Adenosine Triphosphate (ATP) associated with muscular contractions. These events are stress factors that can compromise thermoregulation and significantly affect both pigs welfare and meat quality [1,9,10,31,92]. Warris et al. [40], demonstrated how an increase in temperature in pigs’ ears prior to slaughter at abattoir, had a correlation with the increase in blood temperature in exsanguination. This is very important, since the increase in blood temperature also affects muscle temperature and as a consequence, post-mortem enzymatic reactions are accelerated, affecting pork quality [1–3,5,9,15,40,93].

When combined with the genetic conditions of pigs that carry the recessive ryanodine receptor gene (Ryr1) – known earlier as the halothane gene [14] – those stressors can trigger the physiological, metabolic and behavioural disorders [69] known collectively as porcine stress syndrome [9], a condition characterized by diverse symptomology, including significant hyperthermia. On this topic, Williams et al. [15] evaluated heat production in 5 piglets weighing 45–60 Kg that were susceptible to the fulminant hyperthermia stress syndrome, transported in individual boxes and introduced into a calorimetric chamber. Observations showed that 60% (3) of the piglets adapted to the new environment and displayed normal behaviours, but that 40% (2) began to release high concentrations of CO_2_, increase O_2_ consumption, and dissipate heat via high caloric expenditure. They also manifested reactive behaviours, such as agitation and constant movement. These findings showed that with respect to total heat production in the initial phase (30 minutes), one of these piglets presented over 25.9 J (6–2 kcal) h^−1^ kg^−1ʹ^ and reached 28.25 J (6.76 kcal) h^−1^ kg^−1^ [15].

The skin is the body’s thermoregulating organ *par excellence* because the venous plexuses on its surface regulate blood flow to control and maintain body temperature. Two vasomotor mechanisms regulated by sympathetic vascular innervation explain this principle: a) reflex cutaneous vasodilatation, which activates sympathetic nerves during hyperthermia, causing vasodilatation in the blood vessels in the skin that, in turn, increases blood flow in the periphery so that heat is dissipated towards the environment; and b) reflex cutaneous vasoconstriction, which decreases mean skin and/or internal temperature, thus activating the noradrenergic sympathetic nerves that induce cutaneous vasoconstriction with the consequent reduction of blood flow and conservation of body temperature [94]. When an organism detects the need to adjust its temperature, the systems that regulate cutaneous vasoconstriction, thermogenesis and the reduction of basal metabolism are all activated. In contrast, if it needs to dissipate heat, distinct, species-specific mechanisms will be activated to suppress thermogenesis and cutaneous vasodilatation to eliminate excess heat [94]. With respect to microvasculature, falls in blood pressure occur primarily between arteries and capillaries, which means that the greatest resistance to blood flow takes place in the arterioles. As a result, local blood flow is regulated at the arteriolar level [95] where arteriolar microcirculation adapts to the requirements of individual organs or tissues. Minimal temperature changes are caused by oscillations in microcirculation, and manifested on the surface of the body as heat variations that can be captured by IRT on surfaces 8–14 mm thick. Likewise, disorders of the arteriolar function in systemic diseases that affect microcirculation (e.g. diabetes, hypertension, obesity, metabolic syndrome) have similar patterns of temperature variation in different corporal regions and organs. Microcirculation in the skin, then, is an effective reference for circulation in the body’s main organs [96] since alterations in the microvasculature can be considered precursors of total organ damage.

These processes associated with variations in the temperature of the skin and surface organs that are manifestations of the caloric load in an animal can be monitored during fattening in the pork supply chain, and used as predictive indicators of the adverse effects of the *ante-mortem* period, such as hyperthermia [93]. This is feasible because after death carcases begin to irradiate heat that may reach values of 2.5 J h^−1^ kg^−1^, comparable to the heat dissipated through radiation before vasoconstriction in live animals [15]. Awareness of this may make it possible to prevent defects in meat quality, such as PSE or DFD [93]. In this context, one option for assessing stress through temperature changes in the superficial organs during the various *ante-mortem* stages in fattening pigs is IRT. This is an excellent option because it is a completely non-invasive technique for detecting the intensity of infrared radiation, which correlates directly with temperature distribution in defined corporal regions [96].

## 4. Infrared thermography (IRT)

IRT functions by detecting the temperature of bodies, or specific areas of them. The operating mechanism of IRT involves measuring variations in heat manifested as changes in an organism’s surface temperature. Certain bodily functions, like muscular contractions, generate excess heat that must be eliminated to maintain homoeostasis and the body’s normal functions. Some of the metabolic energy produced in animals and humans is transferred from the generating organ into the environment in the form of heat. Transmission occurs through such basic physical mechanisms as radiation, convection, conduction and evaporation; the latter in the form of sweating or panting [94,97]. IRT operates by detecting and then quantifying surface temperatures in the external millimetres of the dermis [20], where temperature depends on blood circulation to the skin [98]. Body temperature fluctuates constantly throughout the day due to factors that include the physical activity performed by the individual, the physical space where it exists, climatological and environmental conditions, and the presence of infectious processes [94,99].

All conditions that affect blood flow in pigs through processes of regional vasodilatation, hyperthermia, hyperperfusion, hypermetabolism, hypervascularization and hyperaemia can be evaluated by IRT. The surface tissues show areas with infrared emissions generated by high temperatures [100], emissions manifested in the form of colorimetric modifications in thermographic images. IRT has been used to assess animal welfare in several contexts, including evaluating organisms’ responses to the stress factors that can affect production, companion and laboratory animals, respectively [16,17,93,99,101,102]. The search for the optimal site for evaluating temperature by IRT in pigs has led to the testing of the inner ear [40], the orbital region, and the area behind the ear [103]. Other approaches have taken body temperature as a reliable measure of the physiological state of pigs under stressful conditions, such as birthing, when postnatal hypothermia may occur [104], or the stages prior to slaughter [105]. However, Rocha et al. [106] validated this method as an indicator of stress by assessing variations in surface temperature at one of two anatomical locations – the back or rump [105,107,108] – or at multiple anatomical sites [40,103]. To this end, Rocha et al. [106] measured the temperature responses of 120 pigs on the neck, rump, orbital region and area behind the ears under two conditions: gentle handling-induced stress without exposure to stressors *versus* rough handling that included abrupt movements, loud noises and/or physical contact with the personnel. In addition, they evaluated short (30 min) vs. long (90 min) distances walked by pigs and the time travelled (40 min at a loading density of 0.46 m^2^/pig) in a single-deck trailer. To validate the results, they measured such stress indicators as saliva cortisol concentrations, rectal temperature, heart rate and behaviour. Their findings revealed that the sites with the greatest temperature variation and, therefore, the best thermographic indicators were the orbital region and behind the ears, due to the proximity of the eyes and ears to the orbital area of the brain [109]. They concluded that the eyes and ears are metabolic windows that can release the heat stored in the central nervous system which regulates body temperature [110]. These sites also contain an abundant circuit of innervated capillaries of the sympathetic system that quickly respond to changes in blood flow in stressful situations [111,112].

In that same study, Rocha et al. [106], found an increased heart rate during loading in the pigs that received rough handling, and that the pigs subjected to the long walk showed higher saliva cortisol concentrations caused by the physical and emotional stress due to the long distance and high speed of the walk.

Regarding the validation of infrared thermography, these authors concluded that heart rate and saliva cortisol concentrations are highly-correlated with the thermograms obtained from the orbital region and behind the ears. Thus, they argue that these regions dissipate heat quickly through blood flow in the head [113], adding that these zones have lower hair density and a thin layer of fat, so they are closer to both the skin and the bloodstream [114]. Based on these findings, it has been possible to establish the ocular orbit and the area behind the ears as valid sites for performing thermographic evaluations associated with the stress responses that pigs present during handling and transport [106]. However, the low correlation between the temperature obtained from the thermograms and rectal temperature, and the absence of any correlation with the measurements of behavioural indicators also led Rocha et al. [106] recommended that IR be used with other stress indicators to provide a tool for real-time evaluations of the physiological condition of pigs during handling and transport.

Obtaining valid thermographic images requires considering certain key factors:

- the presence of filth or water at the assessment site. This was examined by Banhazi et al. [115] who reported that the presence of water could reduce the precision of the data obtained from the dorsal area of animals.
- the weight of the pigs; at least in newborn piglets, weight affects surface temperature because the heat loss per unit of weight is inversely-proportional to body size (*i.e*., because piglets have a greater surface area, they are more prone to losing heat in a cold environment) [104,116].
- genetic factors: on this topic, Caldara et al. [104] found that piglets with lower surface temperatures came from the same sow, suggesting that genetic factors inherent to dams may affect their piglets’ thermoregulating mechanisms, though they could not rule out the possibility that the heat losses detected were due to soil conduction.
- sensitivity of the image: thermographic images of large groups of animals can reduce sensitivity, since a temperature change in one individual could be masked by the temperature of the group as a whole. Cook et al. [117] found that the spatial distribution of pigs in group images affected the temperature readings obtained; that is, the maximum temperature decreased as spatial distribution increased. This effect can be reduced by using only images in which a few animals cluster together to form a group. Escobar et al. [118] found that only one animal in a group needed to manifest a temperature increase for thermographic camera to detect that variation. This proves the tool’s potential for detecting febrile responses.

**Figure 2.**
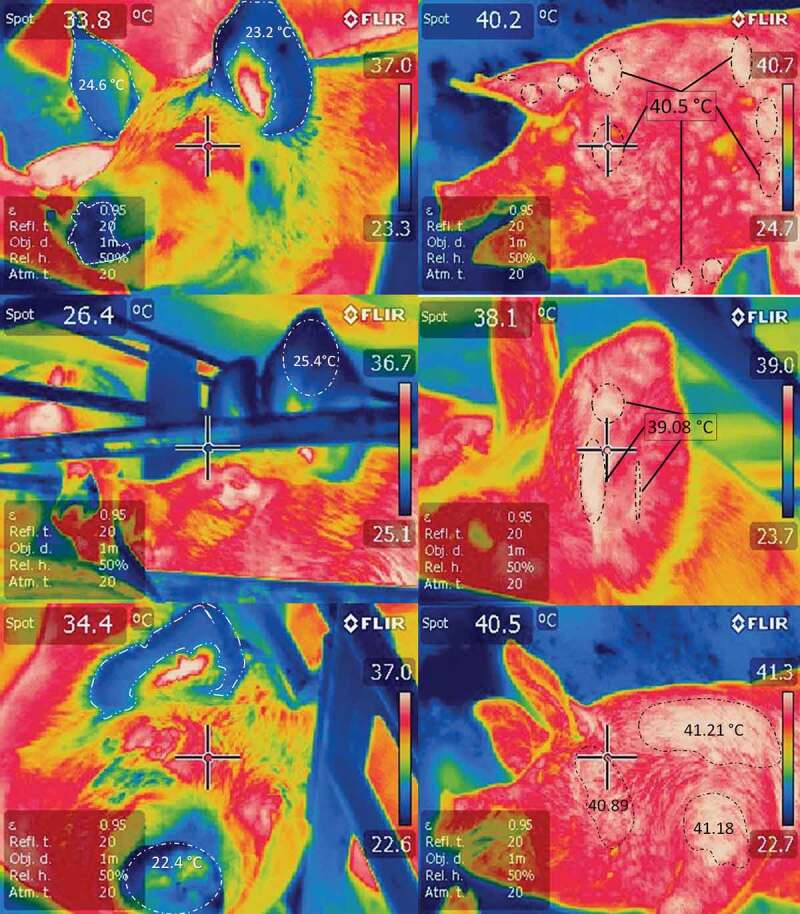
Infrared thermographic changes in pigs upon arrival at an abattoir after transport for several hours. Thermograms on the left are of hogs that arrived at night (coldest time of the day, 18°C) at the abattoir with a journey of 4 hours. Blue areas (highlighted with white dotted lines) are distinguished, with temperatures between 22 and 25°C in peripheral areas such as auricular pavilion and snout; the pigs look fresh. In fact the highest temperatures seemed normal (36°C) in the eye and ear canal areas. In contrast, on the right side of the figure there are 3 thermograms of hogs that arrived at the abattoir at 1:00 p.m. (warmest hour of the day, 34°C), with a journey of 4 hours. White areas (highlighted with black dotted lines) that indicate very high surface temperatures (39 and 41°C) of the loin, face and forearm regions are distinguished. Hogs present hyperthermia, tachypnoea, and in some cases dyspnoea

Finally, the severity of these transport-induced, stress-related disorders has traditionally been evaluated by analysing indices of mortality and the frequency of injuries or fractures, and through behavioural assessments, but other indicators include data obtained from physiometabolic blood profiles and, more recently, the interpretation of infrared thermograms [93]. The utility of thermograms lies in the fact that they can make signs visible that are not detectable through direct observation, as in the case of hyperthermia presented by pigs upon arrival at the abattoir seen in Figure 1, which compares a digital image to a thermographic one. The digital image shows no signs of erythematosus, but the thermographic image reveals white areas that represent temperatures above 40°C, especially around the left ear and the periocular area. As a consequence of this hyperthermic condition, the pig manifested dyspnoea caused by the biochemical processes of hypercapnia and respiratory and metabolic acidosis [31]. IRT can also show the thermal responses of pigs at the end of transport while still in the vehicle (Figure 2). Figure 2 also shows the importance of the duration of the journey in the temperature of the hogs and the usefulness of the IRT to detect changes in the vascular microcirculation of the pig with hyperthermia. Another advantage is that monitoring the body temperature of pigs using a tool like IRT could prevent extreme situations like pork stress syndrome [119–121], characterized mainly by hyperthermia, while reducing the economic losses caused by dead animals [120] or the production of PSE meat [9,22,25,31,119,120]. Lastly, two important elements that must be considered to obtain reliable data and not affect the quality of IRT studies are: ensuring the utilization of high-tech equipment with great sensitivity, and accounting for the large amount of excrement that may cover various body regions of pigs upon arrival at some abattoirs, since when mixed with wet hair this can cause interference [105,107].

## 5. Conclusion

The *ante-mortem* phase of the pork-processing chain constitutes a critical juncture for animal welfare and that it can be measured by metabolic and physiological imbalances that can be captured on the surface of the skin by an infrared thermographic camera. Evaluating the stress that pigs suffer during processing that compromises their welfare has traditionally required invasive techniques; however, IRT is a quick, continuous, effective, non-invasive tool for determining changes in the surface temperature of pigs due to stress during *ante-mortem* stages (loading, transport, unloading, lairage) upon their arrival at the abattoir. Therefore, this technique will allow researchers to detect areas of opportunity to improve animal welfare at critical points during the *ante-mortem* handling of fattening pigs. IRT can also be an effective tool for predicting the repercussions of accumulated stress during the *ante-mortem* phase of the meat-processing chain, and a means of opportunely detecting pork stress syndrome that involves hyperthermia. Finally, the timely evaluation of infrared thermography images of live animals could also prevent defects in meat quality, such as DFD and PSE. However, regardless of the techniques utilized to detect stressors at the critical points of the *ante-mortem* handling of fattening pigs, it is important to implement good processing practices to ensure the welfare of those animals.
